# Long-term remissions of young patients with high-risk follicular lymphoma after first-line autologous stem cell transplantation: Three case reports

**DOI:** 10.1097/MD.0000000000020395

**Published:** 2020-05-29

**Authors:** Rui Lyu, Tingyu Wang, Dehui Zou, Wei Liu, Shuhua Yi, Wenyang Huang, Gang An, Yan Xu, Zengjun Li, Lugui Qiu

**Affiliations:** State Key Laboratory of Experimental Hematology, National Clinical Research Center for Hematological Disorders, Institute of Hematology and Blood Disease Hospital, Chinese Academy of Medical Sciences and Peking Union Medical College, Tianjin, China.

**Keywords:** autologous stem cell transplantation, follicular lymphoma, high-risk, young patients

## Abstract

**Rationale:**

Autologous stem cell transplantation (ASCT) is not routinely recommended as first-line choice for follicular lymphoma (FL). However, we actually have observed that young patients with extremely high-risk factors benefit from ASCT. This study aims to speculate the rationality of ASCT as first-line treatment, through 3 cases and review of the literature.

**Patient concerns:**

3 young-adult patients with FL received ASCT as first-line treatment.

**Diagnosis:**

All the 3 patients were no more than 30 years old and the diagnosis of FL was confirmed by histopathological and immunohistochemical evaluations. They all had multi-organ involvements, and two of them presented with a “leukemic-like” manifestation. Compared with those in the previous literatures, the 3 patients were relatively younger and had more invasive clinical features.

**Interventions:**

The 3 patients received combined chemotherapy plus rituximab, followed by first-line ASCT.

**Outcomes:**

All the 3 patients got complete remission and minimal residual disease negativity after ASCT, The median follow-up time was 109 (97–117) months, and all of them were in remission more than 8 years after transplant.

**Lessons:**

Guidelines for FL are mainly based on elderly patients, but are not suitable enough for all, especially for the young FL patients. For young patients with certain high-risk FL, first-line ASCT does not go against the guidelines, and should be recommended individually.

## 1 Introduction

follicular lymphoma (FL) is 1 of the most frequent indolent Non-Hodgkin lymphomas with the incidence about 3.4 per million a year.^[1]^ The median overall survival (OS) of FL is about 8 to 10 years.^[2]^ Both the guidelines drafted by the National Comprehensive Cancer Network (NCCN)^[3]^ and by the European Society for Medical Oncology (ESMO)^[4]^ recommend R-CHOP (rituximab plus cyclophosphamide, doxorubicin, vincristine, and prednisone) and BR (bendamustine plus rituximab) as standard first-line treatment options in FL. However, autologous stem cell transplantation (ASCT) is not routinely recommended as first-line treatment in the guidelines because of the lack of OS benefit.^[3,4]^ FL is also a heterogeneous disease: most patients always have a favourable prognosis, however, about 20% patients will progress within 24 months and the 5-year survival rate for them is no more than 50%. The ability to accurately risk-stratify patients and then tailor therapy to the individual is an area of ongoing research,^[2,5]^ but the plenty of prognostic factors still do not inform on how best to treat patients, which remains a significant limitation to the practical applications. In this article, we describe 3 FL patients who were very young at the onset. We propose that RCHOP and BR therapies are not always the most appropriate, especially for high-risk patients with aggressive clinical features. Thus, we used ASCT as first-line treatment in the 3 patients, and the effectiveness and rationality of ASCT would be further discussed.

## 2 Patients consent

This study was approved by the ethical committee of Institute of Haematology and Blood Disease Hospital and was conducted in accordance with the Declaration of Helsinki. Patients in this report provided informed consents for the publication of the cases.

## 3 Case description

### 3.1 Case 1

A 29-year-old man presented with enlargement of cervical lymph nodes for 3 months in 2009. The physical examination showed superficial lymphadenopathy and splenomegaly. Complete blood count showed: white blood cells (WBC) 62.18 × 10^9^/L (normal range 4–10 × 10^9^/L), haemoglobin (HGB) 13.7 g/dL (normal range 12.0–16.0 g/dL), platelets (PLT) 143 × 10^9^/L (normal range 100–300 × 10^9^/L). But in 3 days, the WBC count quickly increased to 108.78 × 10^9^/L. Histopathology results of the cervical lymph node confirmed the diagnosis of FL (Grade I-II). The immunohistochemistry (IHC) were positive for CD20, Bcl-2, CD10, negative for CD5, CD3, CyclinD1, and Ki-67 index was as high as 90%. Bone marrow (BM) aspirate smear showed hypercellularity with 62% of lymphocytes and 12% of prolymphocytes. Flow cytometry detection of BM showed positive expression of CD10 and negative expression of CD5. The fluorescence in situ hybridization results confirmed translocation of chromosome 14 and 18, and found the deletion of 17p in 85% of tumor cells. Treatments: Intensive regimens of R-HyperCVAD (rituximab 375 mg/m^2^, cyclophosphamide 300 mg/m^2^ q12 h days 1, 2, and 3, vincristine 2 mg days 4, 11, doxorubicin 50 mg/m^2^ day 4, and dexamethasone 40 mg days 1–4, 11–14) and R-MA (rituximab 375 mg/m^2^, methotrexate 1000 mg/m^2^ day1, cytarabine 2000 mg/m^2^ q12 h days 2, 3) were used for 2 cycles. After 1 cycle, minimal residual disease (MRD) negativity was proved by flow cytometry analysis in both BM and peripheral blood. High-dose CTX (cyclophosphamide 50 mg/kg.d × 2 days) plus granulocyte colony-stimulating factor (G-CSF) were used for mobilization. ASCT was carried out after conditioning with total body irradiation (9Gy, once) and melphalane (140 mg/m^2^). Rituximab maintenance (375 mg/m^2^) was given every 3 months for 8 doses. The patient remains complete remission (CR) and MRD negativity nearly 10 years after transplant.

### 3.2 Case 2

**Figure 1:**
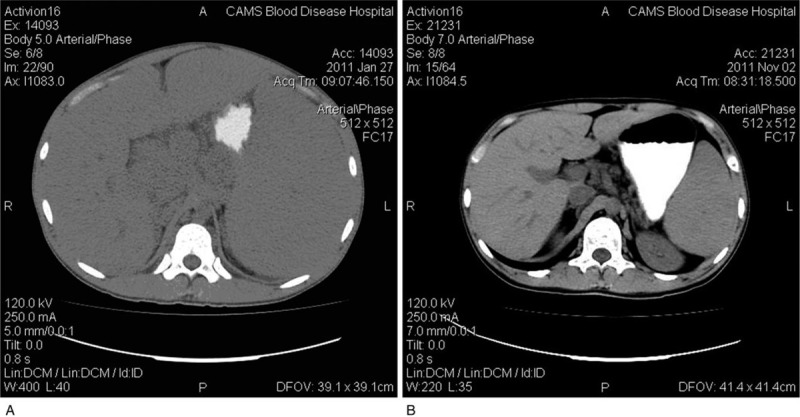
A. Abdomen CT image before treatments in patient 2: enlargements of intraperitoneal lymph nodes, liver and spleen. 1B. Abdomen CT image 3 mo after ASCT in patient 2: intraperitoneal lymph nodes, liver and spleen returned to the normal size. ASCT = autologous stem cell transplantation, CT = computed tomography.

A 19-year-old male college student, presented with abdominal distension for 20 days in 2011. The patient complained of a palpable mass in the left abdominal region, and PE showed a severe splenomegaly (21 cm for the 1st line, 23 cm for the 2nd line, and +9 cm for the 3rd line) and a palpable liver with its lower edge 5 cm below the right costal margin. Computed tomography (CT) scans confirmed the PE findings and discovered enlargements of deep lymph nodes in the thoracic and abdominal cavity (Fig. 1A). The blood routine examination showed: WBC 39.56 × 10^9^/L, HGB 74 g/L, PLT 71 × 10^9^/L. BM smear showed hypercellularity with 55% of lymphocytes. Fluorescence in situ hybridization results found positive for t (14; 18), but negative for the deletion of 17p. Flow cytometry analysis and pathologic evaluation of BM confirmed the diagnosis of FL (grade IIIa). The IHC were CD20+, CD3-, PAX5+, CD5-, CD21 (FDC cell+), CD10+, BCL6-, BCL2+. Treatments: Regimens with R-HyperCVAD, R-FND (rituximab 375 mg/m^2^, fludarabine 25 mg/m^2^ days 1–3, mitoxantrone 10 mg/m^2^ day 1, dexamethasone 20 mg/d days 1–4), and 2 courses of R-EDOCH (rituximab 375 mg/m^2^, etoposide 50 mg/m^2^ days 1–4, doxorubicin 10 mg/m^2^ days 1–4, vincristine 0.4 mg/m^2^ days 1–4, cyclophosphamide 750 mg/m^2^ day 5, dexamethasone 30 mg/d days 1–5) were given sequentially. The patient achieved partial remission (PR) after 4 courses of chemotherapies. ASCT was then carried out after conditioning regimen with R-BEAM (carmustine 300 mg/m^2^ day -7, etoposide 200 mg/m^2^ days -6 to -3, cytarabine 400 mg/m^2^ days -6 to -3, melphalan 140 mg/m^2^ -2d, rituximab 375 mg/m2 days -1, +7). Then, CT scan (Fig. 1B) and BM evaluation proved CR and multiparameter flow cytometry analysis showed MRD negativity. Rituximab maintenance was given with the same schedule as in case 1. The patient is still in remission 8 years after transplant during follow-up.

### 3.3 Case 3

An 18-year-old middle school girl presented with a 3 cm-sized mass in the left preauricular region for one month in 2010. Removal of the mass and pathological examination revealed the diagnosis of FL (grade I-II) and excluded large cell transformation, The IHC were positive for CD20, CD10, BCL2, negative for CD5, and Ki67 index was 30%. 18F-fluorodeoxyglucose (FDG) positron emission tomographies (PET) scan demonstrated high uptakes of FDG in the cervical, axilliary, inguinal regions, and in the thoracic cavity. The standardized uptake values (SUV) were markedly higher than that in the liver tissue, and the largest lymph node was 50mm × 40 mm. BM aspirate showed 22.5% lymphocytes with 1% prolymphocyte, but BM invasion was not proved by flow cytometry and BM biopsy. Treatments: 4 courses of R-CHOP (rituximab 375 mg/m^2^, cyclophosphamide 750 mg/m^2^ day 1, doxorubicin 50 mg/m^2^ day 1, vincristine 1.4 mg/m^2^ day 1, prednisone 100 mg days 1–5) were given and the patient achieved partial remission. The CT scan indicated that there were still lots of enlarged lymph nodes remained, so the other 2 cycles of R-FND were given subsequently. The following PET-CT confirmed CR of the disease, and. then high-dose CTX and G-CSF were used for mobilization. ASCT was carried out with conditioning regimen of R-BEAM, and Rituximab maintenance was not regularly given because of the patient's private reason. The patient remains CR proven by PET-CT 9.5 years after transplant.

### 3.4 Summary of the 3 cases and comparison with the literatures:

The patients’ characteristics are summarized in Table 1, although all of them were no more than 30 years old, they showed the positive expressions of bcl2 and the t (14; 18) chromosome translocation, which could be used to differentiate from paediatric-type FL (PTFL).^[6]^ These patients had multi-organ involvements and also showed lots of high-risk factors which were not included in the conventional FL scoring systems.^[7,8]^ The first patient, who had a high ki67 index (90%) and a high proportion of 17p deletion (85%), was manifested with rapid increase of peripheral blood lymphocytes and short lymphocyte doubling time. The second patient, with the “leukemic-like” manifestation similar to the first patient, showed enormous enlargements of the liver and spleen, which were in accordance with the pathological results of FL (grade IIIa). The third patient with multi-organ involvements, didn’t show the “leukemic-like” manifestation, but had a high baseline total metabolic tumor volume, which was different from the majority of FL patients. Furthermore, by comparison with the 4 former prospective first-line ASCT clinical trials^[9–12]^ in Table 2, we find 3 differences:

(1) There was almost 1000 patients totally enrolled in these trials (n=941), but few were less than 30 years old.
(2) Patients with high FL international prognostic index scores only accounted for 19% to 56% in these trials, and the “leukemic-like” manifestation or high total metabolic tumor volume was not mentioned.
(3) High incidence of second tumor was usually found in these trails, but none was found in our case report.

**Table 1:**
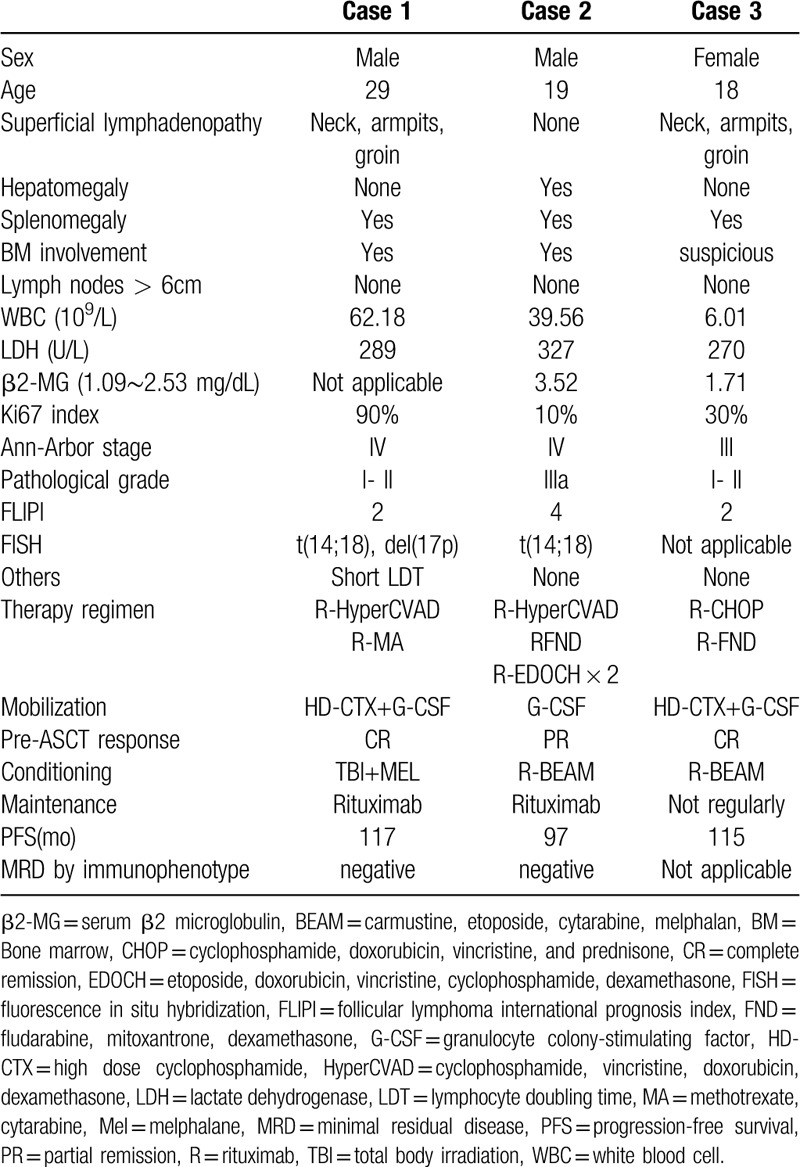
The clinical features and therapy regimens of the 3 young patients with FL.

**Table 2:**
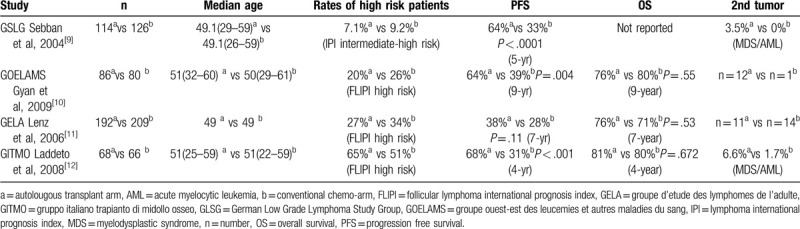
Clinical features, definitions of high-risk follicular lymphoma, efficacies, and secondary tumor incidences from international randomized clinical trials.

## 4 Discussion

Patients with symptomatic and high tumor-burden FL are a very heterogeneous population. The clinical behaviour of the disease ranges from indolent to very aggressive forms, and determines the individualization of treatments. In the era of conventional chemotherapy, the progression free survival (PFS) of FL ranges from 4 to 10 years after the first-line therapy, but is no more than 2 years after the second-line therapy, and is only 1 year after the third and subsequent therapy lines.^[13]^ Therefore, some scholars tried to strengthen first-line treatment so as to avoid the recurrence. Hiddemann et al^[14]^ improved the PFS using GA-101 plus chemotherapy as first-line treatment. Tilly et al^[15]^ explored the combination with lenalidomide and R-CHOP in high tumor-burden FL patients. Recently, progression of disease within 24 months (POD24) has been considered among the most robust predictors of poor survival in FL, however, the definition of high-risk groups at the beginning and what kind of the most appropriate first-line therapy should be adopted are still uncertain.

### 4.1 High risk factors in the 3 patients indicate the early relapse and POD24

According to the literatures,^[16–18]^ high-risk factors in the 3 patients that were outside the conventional FL scoring systems might indicate early relapse of disease and POD24. Kodaira et al first reported that the PFS with leukemic phase in FL at 2 years was 23.4%, versus 73.3% for those without leukemic phase but only manifested with BM involvements,^[16]^ which indicated that “leukemic-like” manifestation was an important predictor for early relapse. O'Shea et al emphasized that the deletion of 17p was another poor prognostic factor associated with FL progression and the median PFS was only 15 months.^[17]^ Meignan et al found in their study that 5-year PFS of high-TMTV patients was only 32.7%,^[18]^ which showed that TMTV was also an important poor predictor for early relapse. Therefore, the 3 clinical features mentioned above predicted early relapse in FL, and the patients with those features would be at high risk of POD24 under conventional treatments.

### 4.2 ASCT should not be abandoned as first-line therapy for the high risk patients

ASCT had been extensively studied in FL. However, its status in first-line treatment was challenged mainly for 3 reasons:

(1) most of the prospective clinical trials^[9–12]^ confirmed that ASCT improved the PFS, but had no advantages in the OS.
(2) Second tumors with high incidence in these trails^[9–12]^ limited the application of ASCT.
(3) Effective salvage therapies after relapse made ASCT redundant in the first-line consideration.^[12]^

However, in our opinion, ASCT in FL may not be in the end of story from the following 5 aspects:

(1) OS did not reflect the actual course of disease and the quality of life, and some patients faced lots of mental stress during the relapse.
(2) Early relapses reduced the chance for second CR and increased the drug toxicities and infections.
(3) Many clinical trials showed that deep-remission patients after first-line ASCT could present with a plateau stage during long-term follow-up,^[10,19]^ which raised the issue of curability in FL.
(4) The occurrence of secondary tumors was minimized through the improvements of conditioning regimens in ASCT.^[20,21]^
(5) POD24 could effectively be reduced in the high-risk patients though ASCT.^[21]^

Based on the above reasons, we think it is too arbitrary to rule out ASCT from the first-line treatment, and ASCT is also in the first-line consideration for FL patients at high risk of early relapse.

## 5 Conclusion

The 3 patients in our report are different from most FL patients. According to the literatures, the high-risk factors would make them relapse very early under conventional treatments, but our results indicate that they get long-term remission through first-line ASCT. As ASCT is not routinely recommended in first-line guidelines, we speculate that recommendations mainly from indolent FL patients may have deficiencies in these patients with such high-risk factors. This report is based on special clinical case and does not deviate from the standard guidelines, and the conclusion from this report must be interpreted with caution and further research is still needed to confirm this finding.

## Acknowledgments

This study was supported by the National Natural Science Foundation of China (81800198), and we thank all our colleagues at the Department of Lymphoma and Myeloma, State Key Laboratory of Experimental Hematology, Institute of Hematology and Blood Disease Hospital, Chinese Academy of Medical Sciences and Peking Union Medical College.

## Author contributions

**Conceptualization:** Rui Lyu.

**Methodology:** Tingyu Wang, Shuhua Yi, Gang An.

**Project administration:** Zengjun Li, Dehui Zou, Lugui Qiu.

**Resources:** Wei Liu, Wenyang Huang, Yan Xu.

**Writing – original draft:** Rui Lyu.

**Writing – review and editing:** Zengjun Li.
